# Distributed Deep Learning in IoT Sensor Network for the Diagnosis of Plant Diseases

**DOI:** 10.3390/s25247646

**Published:** 2025-12-17

**Authors:** Athanasios Papanikolaou, Athanasios Tziouvaras, George Floros, Apostolos Xenakis, Fabio Bonsignorio

**Affiliations:** 1 Department of Electrical Engineering and Computing, University of Zagreb, Unska ul. 3, 10000 Zagreb, Croatia; fabio.bonsignorio@fer.unizg.hr; 2 Department of Electrical and Computer Engineering, University of Thessaly, 38334 Volos, Greece; attziouv@uth.gr; 3 Department of Electronic and Electrical Engineering, Trinity College Dublin, D02 PN40 Dublin, Ireland; florosg@tcd.ie; 4 Department of Digital Systems, University of Thessaly, Geopolis Campus, 41500 Larissa, Greece; axenakis@uth.gr

**Keywords:** distributed deep learning, federated learning (FL), Internet of Things (IoT), sensor networks, plant disease detection, precision agriculture

## Abstract

The early detection of plant diseases is critical to improving agricultural productivity and ensuring food security. However, conventional centralized deep learning approaches are often unsuitable for large-scale agricultural deployments, as they rely on continuous data transmission to cloud servers and require high computational resources that are impractical for Internet of Things (IoT)-based field environments. In this article, we present a distributed deep learning framework based on Federated Learning (FL) for the diagnosis of plant diseases in IoT sensor networks. The proposed architecture integrates multiple IoT nodes and an edge computing node that collaboratively train an EfficientNet B0 model using the Federated Averaging (FedAvg) algorithm without transferring local data. Two training pipelines are evaluated: a standard single-model pipeline and a hierarchical pipeline that combines a crop classifier with crop-specific disease models. Experimental results on a multicrop leaf image dataset under realistic augmentation scenarios demonstrate that the hierarchical FL approach improves per-crop classification accuracy and robustness to environmental variations, while the standard pipeline offers lower latency and energy consumption.

## 1. Introduction

Agriculture plays a vital role in ensuring global food security but remains highly vulnerable to plant diseases that can significantly reduce crop yields and quality [1]. Early and accurate detection of these diseases is vital for allowing timely interventions and minimizing economic losses [2]. Detecting these diseases on time and accurately is essential for preventive action and sustainable crop management. In recent years, the agricultural domain has undergone a significant digital transformation driven by the rapid integration of the Internet of Things (IoT) and Deep Learning (DL) technologies [3].

IoT systems enable the continuous acquisition of heterogeneous data from distributed sensors and imaging devices deployed across agricultural fields. This real-time information enables the intelligent management of critical resources such as water, fertilizer, and energy, while also providing early indicators of plant stress, pest infestation, and disease onset [4]. The fusion of visual and non-visual data, from leaf imagery to environmental and genomic parameters, offers a comprehensive perspective on crop health conditions [5]. Meanwhile, advances in computer vision and DL have emerged as a powerful solution to automatic disease detection, allowing models to learn discriminative visual patterns directly from raw images without manual feature engineering [6]. These approaches have shown excellent performance in tasks such as fruit classification, weed identification, and crop yield prediction, substantially improving accuracy and reducing the time required for manual inspection [7]. However, most existing DL systems are based on centralized computation approaches and static datasets, which limit their applicability in dynamic and distributed agricultural environments.

As a result, there is an increasing number of research works that try to fuse the IoT and DL methodologies for agricultural monitoring and disease prediction [8]. Moreover, recent advances have introduced edge and fog computing frameworks to bring inference closer to data sources, reducing latency and communication costs [9]. As a result, Federated Learning (FL) has emerged as an effective distributed learning paradigm that enables multiple devices to collaboratively train a global model without sharing their local data, preserving data ownership and privacy [10]. However, the adoption of FL in agricultural applications remains limited, even though integration of FL with edge devices can enable real-time decision-making and significantly reduce latency in agricultural IoT environments [11].

In this paper, we propose a distributed DL framework using FL for the diagnosis of plant diseases in IoT sensor networks. The main contributions of this paper are summarized below:

- *First*, we design and implement a fully distributed deep learning architecture that integrates IoT sensor nodes and an edge computing node using a federated learning paradigm. Each IoT node performs local model training on plant image data, while the edge node aggregates model updates through the Federated Averaging (FedAvg) algorithm to create a global model without requiring data centralization.
- *Second*, we introduce and evaluate two complementary FL training pipelines, a standard single model approach and a hierarchical approach combining a crop classifier with crop-specific disease models.
- *Third*, we present a detailed power consumption model for heterogeneous IoT nodes, comparing CPU-only and GPU-enabled devices, and quantifying the computational costs of each training configuration.

Finally, we perform extensive experiments on a multicrop leaf image dataset under multiple realistic augmentation scenarios that emulate field conditions such as variable illumination, focus, and compression. The results demonstrate that the hierarchical pipeline improves per-crop classification accuracy and robustness, while the standard pipeline provides reduced latency and power consumption suitable for resource constrained IoT devices.

The remainder of this paper is organized as follows: Section 2 highlights recent developments in deep learning and federated learning for agricultural and IoT-based applications. Section 3 presents the theoretical background of the proposed approach, focusing on the fundamentals of distributed and FL techniques. Section 4 describes the main contribution of the paper, detailing the IoT and edge computing layers, the FL pipeline, and the power modeling of IoT nodes. Section 5 outlines the experimental setup, including the dataset description, training procedure, and evaluation metrics. Section 6 presents our experimental results, which are followed by conclusions in Section 8.

## 2. Related Work

In this section, we briefly review previous research related to deep learning and IoT-based techniques developed for smart agriculture and plant disease detection. Recent advances in DL and IoT technologies have raised a wide range of approaches with regard to intelligent and data-driven agriculture [12].

More specifically, the authors in [13] applied Convolutional Neural Networks (CNNs) to predict crop yield using multispectral and RGB images captured by Unmanned Aerial Vehicles (UAVs). Their approach demonstrated that CNN-based models can achieve high accuracy in estimating biomass and yield, particularly when RGB data are used during early growth stages. Similarly, authors in [14] created a taxonomy separating existing studies into classification-based and object detection-based approaches and evaluated several state-of-the-art models in the PlantDoc dataset [15]. Additionally, experimental results showed that YOLOv5 [16] achieved the highest accuracy for object detection, while ResNet50 [17] and MobileNetV2 [18] offered the best balance between accuracy and computational efficiency. Although these works demonstrated remarkable results in image-based applications, they rely primarily on centralized data processing and high-performance computing environments, which limit their scalability in real-world field deployments.

Apart from the DL approaches, several works focus on developing IoT-driven systems for real-time plant disease detection and environmental monitoring. Authors in [19] proposed an IoT-based cognitive monitoring framework designed to forecast early plant disease outbreaks by continuously collecting soil and environmental data through wireless sensor networks. The proposed system integrated artificial intelligence algorithms to emulate expert decision-making and issue early warnings for higher quality yields. In addition, the authors in [20] introduced an intelligent solution enabled by IoT for the detection of leaf disease that combined temperature, humidity and soil moisture detection with image-based disease recognition using a Raspberry Pi controller and camera interface. Furthermore, the authors in [21] developed an IoT-enabled detection and classification system for banana leaf diseases using image processing and random forest classification. The proposed platform achieved nearly 99% detection accuracy, demonstrating efficiency in lightweight learning models deployed at the edge for agricultural applications. Despite their effectiveness in localized environments, these IoT-based systems do not incorporate advanced or adaptive DL techniques capable of handling diverse field conditions, crop varieties, or environmental variations. Most rely on static models and single-node processing, which limits their applicability in large, heterogeneous agricultural environments.

The approach in [22] bears a resemblance to the proposed method, since an FL strategy is employed for distributed crop yield prediction, using the Federated Averaging (FedAvg) algorithm. In that work, deep residual networks (ResNet-16 and ResNet-28) are trained across decentralized datasets collected from multiple agricultural sites. However, these models require substantial computational resources and memory due to the complexity of residual architectures, making them unsuitable for deployment on resource-constrained IoT nodes. In contrast, the proposed framework adopts an EfficientNet-based model, which offers a more favorable trade-off between accuracy and computational efficiency, enabling distributed learning on low-power IoT sensor networks.

## 3. Background

In this work, we make use of an FL method, which is considered a subcategory of the larger group of distributed learning [23]. In this section, we present the preliminaries of the FL technique, and we analyze the DL model we use for the training and inference operations.

**Federated Learning.** Generally, FL algorithms consist of two distinct phases: (i) a local model training phase; and (ii) a global model aggregation phase. During the local model training, each *i*-th node independently trains a DL model ($M_{i}$), using as training set the locally stored data. This process is repeated for a predetermined number of epochs. In the sequel, the global model aggregation phase kicks in, where an edge computing node (ECN), acting as coordinator, collects all the trained $M_{i}$ models from the local *i* nodes. Then, it aggregates the collected models into a global $M_{g}$ model, an action that marks the completion of an FL round. Finally, the $M_{g}$ is broadcasted back to each node *i* to restart the local model training process. This loop between local model training and global model aggregation is repeated for a predefined number of rounds. Below we elaborate on the details of each phase.

**Local model training.** In FL environments a number of autonomous nodes are deployed over a geographical area. Each node *i* is able to train a DL model to fit the locally stored data, which is collected by its surrounding environment. Without a loss of generality, we assume that DL models are composed of several layers. Each layer utilizes the following equation to generate its output:

(1) $$f^{k}=\sigma^{k}\left(W^{k}\cdot X^{k-1}+b^{k}\right)$$

For the *k*-th layer under investigation, $\sigma^{k}$ represents its activation function, $W^{k}$ its weight matrix, $X^{k-1}$ indicates the output of the previous layer k−1, which is used as input for the current layer *k*, and $b^{k}$ is the layer’s bias. Notably, the operation between $W^{k}$ and $X^{k-1}$ can take many forms, such as 2D convolution, attention, de-convolution, depth-wise convolution and so on. Upon the final layer, which is also the model’s output, is applied a loss function that designates the error rate of the model. In supervised training operations, the loss function can be calculated using the following formula:

(2) $$L\left(y,\hat{y}\right)=L\left(y,W^{z}\right)$$

where $\hat{y}$ is the label prediction of the model, $W^{z}$ is the output of the final model layer and *y* is the ground truth, i.e., the true label for the current sample. *L* is the function that represents the loss function itself, such as MSE, cross-entropy, categorical cross-entropy, hinge, tangent and so on.

After the loss value is calculated for each sample, each weight matrix $W^{k}$ of each layer *k* is updated in order to converge to the target *y* value, using the back-propagation process, as follows:

(3) $$W_{new}^{i}=\left(1-\eta \lambda \right)W_{old}^{i}-\eta \frac{\partial L(y,\hat{y})}{\partial W_{old}^{i}}$$

where η is the learning rate, which is set by the user, λ is the weight decay parameter that is often used to reduce overfitting, and the term $\frac{\partial L(y,\hat{y})}{\partial W_{old}^{i}}$ represents the gradient (partial derivative) of the loss function with respect to the weights $W_{old}^{k}$ of the layer *k*. This gradient is computed as follows:

(4) $$\frac{\partial L(y,\hat{y})}{\partial W_{old}^{i}}=\left(\prod_{n=1}^{n=k}\frac{\partial \sigma^{k}}{\partial W_{old}^{i}}\right)\frac{\partial L(y,\hat{y})}{\partial \sigma^{n}}$$

The equation presented above essentially captures the chain rule that the back-propagation process follows. This product represents the gradient for each layer, designating how much the weight values should change in order to better fit the input data. The local model training process is conducted iteratively for each node for a number of epochs set by the user. After this operation is completed, all nodes *i* send their trained models $M_{i}$ to the ECN for aggregation.

**Global model aggregation.** The ECN, after collecting the locally trained $M_{i}$ models, aggregates their weights to formulate one global model $M_{g}$. This process can be conducted using several aggregation techniques such as FedAvg [24], FedProx [25], FedNova [26], Scaffold [27], MOON [28], etc. One of the most commonly used methods, FedAvg aggregates the $W_{i}$ weights using the following formula:

(5) $$W_{g}^{k}=\sum_{a=1}^{a=i}W_{i}^{k}$$

where the $W_{g}^{k}$ is the new aggregated weight matrix of the *k*-th layer of the model $M_{g}$, *i* is the total number of local models collected by the ECN, and $W_{i}^{k}$ is the weight matrix of the *k*-th layer for the local model *i*. As a result, the global model $M_{g}$ consists of the newly aggregated layers $W_{g}$, as in [29].

When this operation finishes, a training round is completed. After this, the ECN broadcasts the global model $M_{g}$ to each local node *i* to resume the local training process. These tasks, in turn, will be completed after the predefined number of epochs is reached, and the nodes will send the models $M_{i}$ back to the ECN for aggregation. The FL training procedure is finished when the maximum number of training rounds is reached.

**Figure 1:**
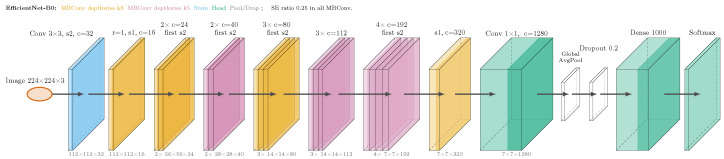
The EfficientNet-B0 architecture, which is deployed within this work.

**Deep Neural Network model.** In this work, we leverage the EfficientNet-B0 model, as proposed by previous research in [30]. The architecture of EfficientNet-B0 is illustrated in Figure 1 and is composed of a series of Mobile Inverted Bottleneck Convolution (MBConv) layers. MBConv layers essentially use depthwise separable convolution operations, which greatly improve the computational complexity of the model. Thus, in each layer, the input features are first expanded via the application of depthwise convolution, and then a pointwise convolution operation is invoked to project them back into a narrower dimension. Finally, the output of most layers is determined by a squeeze-and-excitation block, which leverages the attention mechanism to selectively amplify informative features. In our experiments, we distribute the EfficientNet-B0 model to the corresponding nodes, which train it using locally stored data. After the local training operation is completed, the individual local models are collected by the ECN to perform the global model aggregation, similarly to the process described within this section.

We opt to conduct our experiments using the EfficientNet-B0 model, since it is widely used in federated environments, as indicated by previous research in [31,32,33]. Generally, EfficientNet-B0 is a lightweight model, ideal for IoT applications, which performs well in image detection and classification problems. We should also note that the methodology proposed in this work is model agnostic, and thus, it is compatible with any kind of Deep Neural Network (DNN) model.

## 4. Methodology

### 4.1. System Architecture

**Figure 2:**
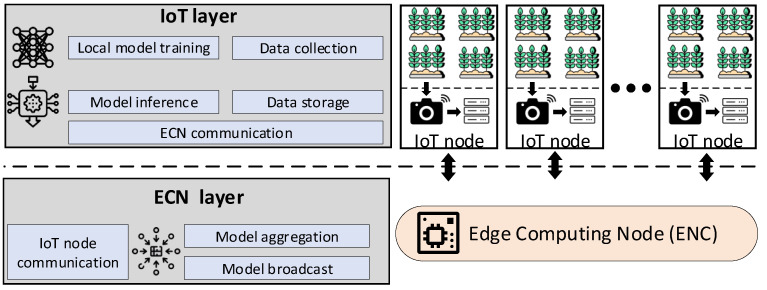
The overall system architecture that consists of a number of IoT nodes used for monitoring and local processing tasks and of an ECN that aggregates the locally trained DNN models.

Figure 2 illustrates the overall system architecture we employ in this work. We consider a variable number of IoT nodes deployed over a geographical area and one edge computing node (ECN), which is deployed at the edge of the network. Thus, our system architecture is composed of two layers: (i) an IoT layer and (ii) an ECN layer.

The IoT layer contains several autonomous IoT nodes, each one of which can perform the following tasks:

- **Data collection:** Each IoT node is equipped with a camera and is able to take photos in real-time from its surrounding environment. In this work, we deploy a distributed learning method for agricultural applications, and thus, we consider such images to capture different types of plants. Nonetheless, this method is generalizable to other types of data as well.
- **Data storage:** Each IoT node is able to locally store the data collected (in our case the plant images). As a result, each node *i* can formulate a local dataset $D_{i}$ that can be used to train a local DNN model.
- **Local model training:** Each IoT node has the computational capacity to train a DNN model $M_{i}$ that fits the locally stored dataset $D_{i}$.
- **Model inference:** IoT nodes can utilize their trained models $M_{i}$ to perform inference (i.e., model testing) upon the collected data.
- **Communication with ECN:** Each IoT can communicate only with the ECN and is able to send and receive information. IoT nodes cannot communicate between themselves, since this would require a more complex communication infrastructure, which is unfit for sensor networks.

The ECN layer contains one ECN, that is able to execute the following tasks:

- **Model aggregation:** ECN can collect and then aggregate the $D_{i}$ models from the corresponding IoT nodes. This process results in a global $M_{g}$ model that is broadcasted back to the IoT nodes. In this paper, we use the FedAvg technique for the model aggregation operation, as in [24].
- **Communication with IoT nodes:** ECN can establish a bidirectional communication channel with each IoT node individually.

In the following sections we leverage the system architecture described above to deploy our methods and to conduct the evaluation process.

### 4.2. Design of Federated Learning Pipeline

**Figure 3:**
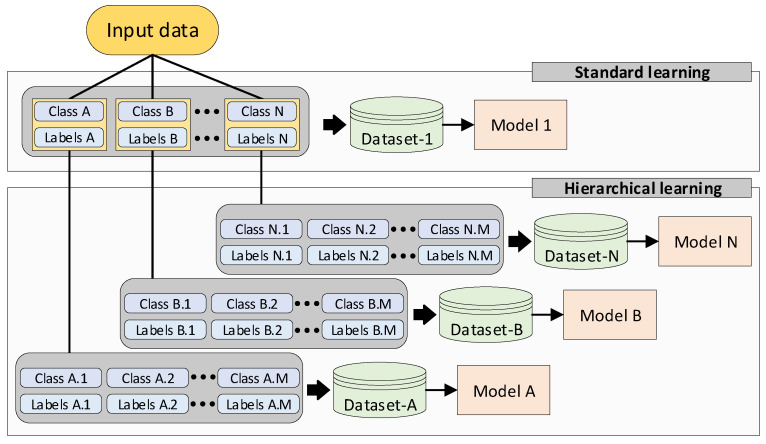
The standard and hierarchical learning strategies, which are used in this work.

In this work, we design two different FL pipelines capable of handling both standard learning and hierarchical learning methods, as illustrated in Figure 3.

**A standard learning pipeline** is developed. Standard learning refers to the prevalent machine learning techniques under which a collection of input data from different classes A−N, where each class has a unique label A−N, is used to formulate a training dataset (dataset-1 in Figure 3). Then, a portion of this dataset is leveraged to train a DL model, while the rest of the data is used as a test set for validation purposes.

**Hierarchical learning pipeline** is developed. Hierarchical learning is a different machine learning strategy that breaks down complex input data into a hierarchy of simpler datasets [34]. This strategy has proven to achieve better results in domains where input data is diverse [35], or when the problem can be divided into concrete sub-tasks [36]. In this work we apply this strategy by breaking down the dataset that was generated by the standard learning method into smaller *N* datasets. Each dataset 1,2,3,⋯,N is essentially a subset of the dataset-1 and contains its own classes *M* and labels *M*. As a result, instead of training 1 DL model, the hierarchical learning approach trains *N* models, using *N* different datasets.

**Figure 4:**
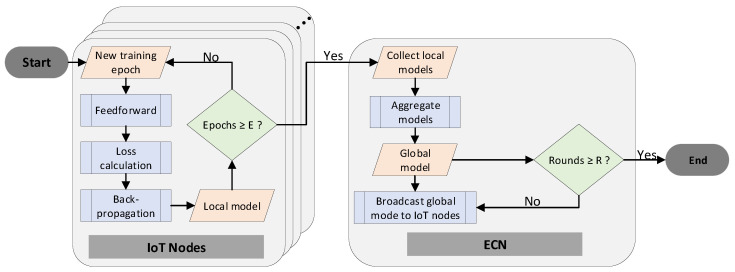
The federated learning pipeline that is used in this work for each dateset generated by the standard and hierarchical learning methods.

The model training process, which is deployed upon the proposed FL pipelines, is depicted in the flowchart of Figure 4. We should note that this procedure is conducted once (for the 1 model), when the standard learning strategy is in place, or *N* times (for *N* models), in the case of hierarchical learning operations.

In FL environments, each IoT node trains locally and independently its own DL model, using its own data as the training set. This local training operation consists of several epochs, with each epoch containing the following phases: (i) a feedforward phase where the input data is propagated within the DNN; (ii) a loss calculation phase where the DNN model output is compared with the ground truth (label), and the loss value is generated; and (iii) a back-propagation phase in which the DNN’s weights are updated with respect to the loss value. After a predefined number of epochs (*E* in our case) has been reached, the local model training is completed. In the sequel, all local models are dispatched to the ECN. The ECN, after collecting the IoT models, starts the model aggregation process, as described in Section 3. This operation outputs a single global model and marks the end of a training round. If a predefined number of training rounds has been achieved (*R* in our case), the FL training is completed, and the global model is the output of the training operation. On the other hand, if the number of training rounds is smaller than *R*, the FL process continues. To this end, the ECN broadcasts the global model to each IoT node, where a local model training operation initiates again.

### 4.3. Power Modeling of IoT Nodes

Efficient energy utilization is an important factor in the deployment of distributed IoT sensor networks, particularly for FL environments where each node performs local computation during training and inference [37]. In this subsection, we present the hardware characteristics of the IoT nodes considered in our system and describe the methodology adopted for estimating their power consumption through benchmarking and software profiling. In our approach, we consider two types of IoT nodes, which are modeled according to their hardware capabilities as follows:

- **CPU-only node:** This node features an ARM-based processor without CUDA acceleration. It performs on-device inference and participates in local FL training using only CPU resources.
- **GPU-enabled node:** This node integrates an ARM CPU with a CUDA-capable embedded GPU (e.g., an NVIDIA Jetson-class device). It supports both inference and local FL training, benefiting from parallelized tensor operations.

These two configurations represent realistic deployment scenarios in heterogeneous agricultural IoT networks, where energy and computational capacities can vary across devices.

Let $P_{\mathrm{dev}}$ denote the instantaneous power consumption of a given IoT node. In general, the total energy consumed by a computational task can be expressed as

(6) $$E=\int_{0}^{T}P_{\mathrm{dev}}\left(t\right)\;dt$$

where *T* represents the execution time of the task. In most IoT systems, the power profile remains approximately constant during short inference or training cycles. Thus, the average energy consumed by a computational stage can be approximated as

(7) $$E_{avg}=P_{\mathrm{dev}}\cdot t_{\mathrm{stage}}$$

where $t_{\mathrm{stage}}$ denotes the time required to execute the stage. This formulation captures the relationship between device power, processing time per stage, and average energy expenditure for a single computational task performed on an IoT node. For a dataset of *N* images, the total energy consumed by a device is

(8) $$E_{total}=N\cdot E_{\mathrm{avg}}$$

If multiple IoT devices share the workload; the total energy should be divided according to the number of images processed by each device. Typical embedded configurations are characterized by approximate power profiles of:

(9) $$P_{\mathrm{CPU}}=3.0\;W,\;P_{\mathrm{GPU}}=5.0\;W,$$

corresponding to CPU-only and GPU-enabled nodes [38,39], respectively. These constants provide a theoretical baseline for estimating the energy requirements of different hardware platforms in the distributed network. In the proposed framework, two processing pipelines are defined to account for the structure of the learning tasks as follows:

- **Standard learning pipeline:** a monolithic single-head model (EfficientNet-B0) trained to classify all disease classes across crops.
- **Hierarchical learning pipeline:** a modular structure consisting of a crop router that identifies the plant type and a corresponding crop-specific disease classification head.

Finally, the total energy consumption for each configuration can be calculated as

(10) $$E_{\mathrm{standard}}=E_{\mathrm{total}},\;E_{\mathrm{hierarchical}}=E_{\mathrm{router}}+E_{\mathrm{disease}}$$

where $E_{\mathrm{model}}$ is equivalent to the per-stage energy, $E_{\mathrm{router}}$ is the energy for the crop identification stage, and $E_{\mathrm{disease}}$ is the energy for the disease classification stage once the crop is known. This decomposition allows the analysis of energy distribution across model components, enabling an assessment of the trade-off between model complexity and energy efficiency. In an FL environment, local training and communication stages contribute to the overall energy consumption of each node. For node *j*, the total energy consumed across *R* global communication rounds can be expressed as

(11) $$E_{j}^{\mathrm{FL}}=R\cdot \left(E_{j}^{\mathrm{comp}}+E_{j}^{\mathrm{comm}}\right)$$

where $E_{j}^{\mathrm{comp}}$ represents the local computation energy, and $E_{j}^{\mathrm{comm}}$ corresponds to the communication energy associated with model parameter exchanges.

## 5. Experimental Setup

In this section, we present the experimental setup employed in our study, focusing on datasets, the federated learning training process and the evaluation metrics used to assess model performance. Particular attention is given to selecting software tools and libraries to support the distributed deep learning process, efficient data handling and performance monitoring. The combination of such components established a reliable computing framework for implementing model training and evaluation across multiple agents.

Going into technical details, the implementation was developed using Python 3 [40] as a core programming language, due to its extensive ecosystem for ML computing. The components for DL model and inference were implemented with PyTorch 2.9.0 [41], which provides an efficient and flexible framework. To handle computer vision tasks, we employ Torchvision [42], offering utilities such as the pre-trained EfficientNet-B0 architecture [30], as well as the ImageFolder dataset loader and various data transformation utilities. For the federated learning orchestration, we apply Flower (flwr) framework [43], enabling distributed model training across multiple agents.

As far as model evaluation metrics are concerned, we use scikit-learn [44] to compute accuracy and macro-F1 score. Image operations and data augmentations are handled by Pillow (PIL) [45], while NumPy [46] facilitated efficient numerical operations and the storage of aggregated model weights in .npz format. In addition, psutil [47] is applied to measure process memory consumption in terms of resident set size (RSS). Finally, several modules from the Python standard library, such as time, csv, json, os, and multiprocessing, are employed for timing, data serialization, file management, and parallel processing, respectively.

### 5.1. Dataset and Use Cases Description

In our study, we employ an RGB leaf image dataset, labeled at the level of crop—disease pairs. Each image is associated with a single class, resulting in a total of 38 distinct labels covering the following crops: *apple, blueberry, cherry, corn, grape, orange, peach, pepper, potato, raspberry, soybean, squash, strawberry, and tomato.* As an example of the label granularity, the apple category includes the following classes: apple_scab, black_rot, cedar_apple_rust and healthy). All images are resized to 224×224 pixels prior to training and evaluation in all experimental configurations.

**Figure 5:**
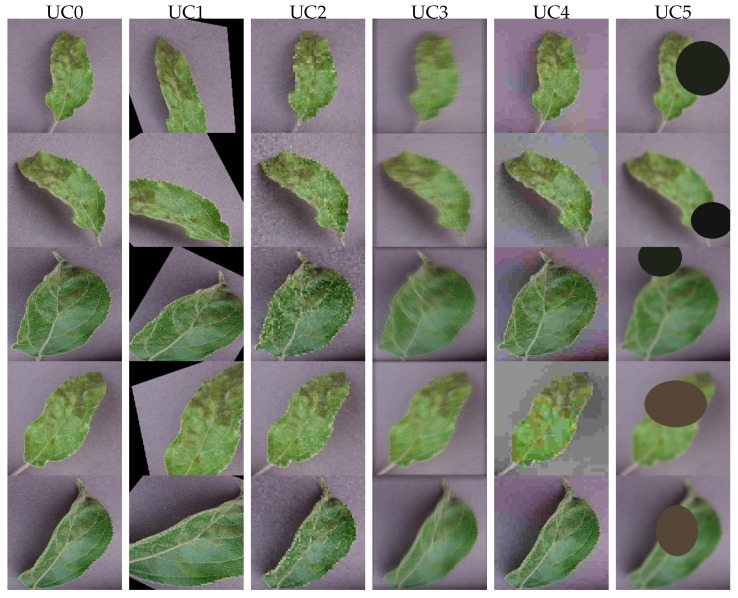
Original and five augmentations per sample (left→right).

To better approximate real-world field conditions and assess model robustness under practical variations, such as illumination, viewpoint, focus, compression, and framing, a data-augmented version of the dataset was created. For each class, images are split into 5 equal parts, and one recipe is applied to each class, so every class keeps the same number of images, and the 5 variants are all balanced (20% each). To this end, we highlight the following 6 Use Cases (UCs) as depicted in Figure 5, on which we base our papers’ comparisons:

- **UC0:** The *Lab Environment* UC refers to the original dataset, untampered images, which are the baseline for comparisons.
- **UC1:** The *SunnyAngle* UC refers to an environment with the following characteristics: *slight perspective tilt, small rotation, mild brightening/contrast, soft shadow; bright and strong sunlight, oblique camera angles, and leaf or hand shadows in the field.*
- **UC2:** The *OvercastNoise* UC refers to an environment with the following characteristics: *darker, lower contrast, slight desaturation, Gaussian sensor noise; mimics cloudy or dusk conditions with higher camera ISO and muted colors.*
- **UC3:** The *Defocus* UC refers to an environment with the following characteristics: *mild blur with small zoom jitter and rotation; motion-blurred image, wind-driven motion, or shallow depth-of-field misfocus.*
- **UC4:** The *JPEGandCast* UC refers to an environment with the following characteristics: *JPEG re-compression artifacts and warm/cool color cast; mimics on-device compression, messaging/export pipelines, and automatic white-balance drift.*
- **UC5:** The *OffCenter* UC refers to an environment with the following characteristics: *off-center crop, re-centering, light exposure/contrast changes; mimics imperfect framing from mobile mounts or partial leaf capture.*

For each Use Case, we distribute the data among the available IoT clients in a uniform fashion. Each client formulates a local dataset with the corresponding number of classes (depending on the FL pipeline scenario, as described in the following section). We randomly split the data among the corresponding classes, making sure that each class contains the same number of images. Using this method, we formulate Independent and Identically Distributed (i.i.d.) datasets for each client.

### 5.2. Federated Learning Training Procedure

This subsection describes the practical implementation of the federated learning process, emphasizing the two training pipelines evaluated in this study; the interaction between clients and coordinating ECN, the sequencing of training tasks, and the initialization and parameter-sharing strategies employed across models. In all experiments, the optimizer, batch size, learning rate schedule, label smoothing, dropout, and data augmentation settings were kept constant across runs to ensure that any observed performance differences arise solely from the organization of the label space and the scheduling of training, rather than from variations in hyperparameters. The two training pipelines are as follows:

**Pipeline A**: *Standard training:* In this standard setting, we train an EfficientNet-B0 classifier, that predicts directly over all disease labels in this study (38 classes in total). The edge-cloud coordinator initiates a single federated job, broadcasts the current global weights to the participating 10 IoT clients, at the beginning of each round, and collects updated weights after a fixed local training of 5 epochs on each device. Aggregation is weighted by the number of local training samples per client, and the resulting global model is redistributed to start the next round for 10 rounds in total. This pipeline minimizes orchestration complexity and produces a compact deployment footprint, since inference requires only one model regardless of crop.

**Pipeline B**: *Hierarchical training:* To reduce cross-crop misclassifications and to better reflect deployment scenarios in which individual sites typically encounter a limited range of crops, the learning process is decomposed into two distinct model families, each trained as an independent federated task. Firstly, we train a crop classifier on the complete dataset to identify the crop category; this job includes all available clients because every image contributes a crop label. Secondly, we train one disease classifier per crop; for each crop subset, we filter the dataset to that crop only, launch a new federated job, and enroll only clients that hold local samples of that crop in their filtered split. Each per-crop disease model is thus optimized independently and exclusively on relevant data, and parameters are never averaged or shared across different crops during aggregation. During inference, an input is first assigned a crop, by the crop classifier, and only the corresponding crop-specific disease model is invoked to predict the final disease label.

All federated training tasks follow a common synchronous routine. In each round, the ECN broadcasts the current model weights to all participating clients, which then perform local training for a fixed number of epochs. Upon completion, the ECN aggregates the model updates received before a predefined timeout, while disregarding contributions from delayed or failed clients for that round. Training tasks are scheduled sequentially to manage bandwidth usage and prevent interference between concurrent jobs. Specifically, the crop classification model is first trained to convergence, followed by the sequential training of the crop-specific disease models, each executed as an independent federated session. A client participates in a hierarchical (crop-specific) training session only if its local data shard contains at least one sample of the corresponding crop; otherwise, it is excluded from that session. This participation policy prevents degenerate updates originating from irrelevant data and ensures that communication is restricted to clients whose local observations align with the target crop.

Both pipelines use the same EfficientNet-B0, with the same parameters as shown in Table 1, as a backbone to ensure comparability. The standard learning classifier is initialized using the same pre-trained weights used throughout the study. In the hierarchical pipeline, the crop classifier is trained first using the shared backbone, while each per-crop disease model is initialized from the same pre-trained backbone and equipped with a classification layer sized to the diseases of that crop; classification layers are freshly initialized for each head. No cross-head parameter mixing occurs at any point: the crop classifier and all per-crop disease models maintain separate federated training states and separate global checkpoints.

The standard training pipeline capitalizes on maximal data pooling and is simple to deploy on constrained devices that must carry a single network. The hierarchical design confines decision boundaries within a single crop, enabling smaller and more specialized heads that can be updated independently when a specific crop changes or expands, while leaving unrelated models untouched. Presenting both allows practitioners to choose between a one-model solution with minimal coordination and a crop-aware solution that trades a modest increase in orchestration for reduced inter-crop confusion and targeted updates in the field.

### 5.3. Evaluation Metrics

To compare the *standard* and *hierarchical* pipelines on equal footing, we adopt a common set of measurements, collected during training and during summation, keeping their definitions identical across pipelines. To this end, differences observed later, reflect the organization of the models, rather than the way they are measured. As far as task performance is concerned, we compute overall accuracy on the held-out test split together with macro-averaged precision, recall, and F1, and we complement these aggregates with per-class F1 and confusion matrices to expose class imbalance and recurrent confusions that a single score may conceal. In the hierarchical pipeline case, we treat the end-to-end disease label produced by the crop classifier, followed by the selected crop-specific head, as the primary outcome, and we additionally separate the problem into crop identification accuracy and conditional disease performance on correctly routed samples; this decomposition makes explicit whether benefits arise from specialization within a crop or are offset by routing mistakes.

Given that training is conducted in a federated setting, model behavior is monitored across clients and communication rounds by systematically recording, after each aggregation, the local test accuracy and macro-F1 score for every participating client. These metrics are then summarized as both a sample-weighted average, representing the global learning objective, and an unweighted average, capturing site-level fairness. In addition, the training loss reported by individual clients and the aggregated loss computed at the ECN are recorded to facilitate performance tracking over time. The resulting statistics and trends are visualized as plots in the following section. Communication overhead is measured as transmitted bytes per round and in total, separated into uplink and downlink, by multiplying the serialized model or update size with the number of broadcasts and uploads actually performed. For the hierarchical pipeline we attribute these bytes to the crop classifier job and to each crop-specific job before also reporting their sum, which enables a like-for-like comparison with the single-model alternative and clarifies whether additional orchestration materially increases the communication budget.

Finally, to reflect deployment constraints, we evaluate runtime and resource use with our inference procedure under the device profiles introduced in the power-modeling subsection. For each model, we report per-image latency, throughput, peak RAM, and, when applicable, peak GPU memory. Moreover, we compute energy per inference by integrating device power over the inference window and additionally report the corresponding average power per image, allowing us to separate the influence of inference duration from that of device power consumption. In the hierarchical pipeline we provide both the end-to-end cost, crop classifier plus selected head, and the isolated cost of each component, and we include the on-disk model size as an indicator of storage and update burden.

## 6. Experimental Evaluation

**Figure 6:**
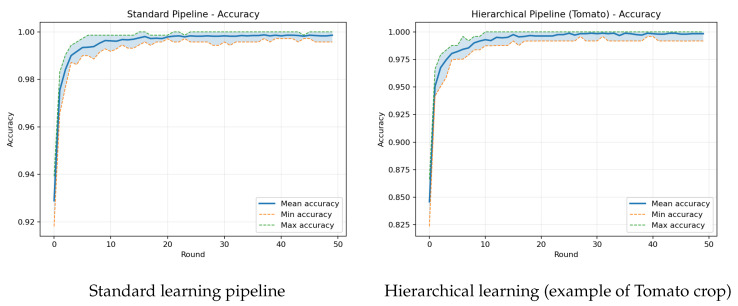
Training–validation accuracy per client for both models.

This section presents the experimental results regarding the two aforementioned pipelines, trained on the original dataset and evaluated on the augmented test set, as previously described. Our goal is to quantify robustness under a realistic domain shift. In essence, data acquired in the field rarely match the curated conditions of training. Hence, an appropriate model that preserves accuracy on augmented inputs is more likely to sustain performance during deployment. We present the training–validation accuracy curves per client for the global FL model and, for the per-crop model, the Tomato example (Figure 6), a per-class results table comparing the two pipelines (Table 2), and a device comparison table summarizing end-to-end accuracy together with image processing time, peak RAM, the average power per image during the inference window, and on-disk model size per image (Table 3).

To contextualize the per-image power and energy values reported in Table 3, we note that the full test workload contains 7156 images, which are evenly distributed across the 10 participating IoT nodes. While Table 3 reports the average power per image, a time-normalized metric derived from the short inference window, the total energy consumption of each device must be computed over the full number of processed samples. More specifically, if device *j* processes $N_{j}$ images, its total inference energy is given by (8), where both $P_{dev}$ and $t_{stage}$ are obtained by Table 3. In our setup, each device handles approximately 716 images, allowing the small per-image energy values to accumulate into a realistic total energy footprint. Importantly, the resulting aggregate energy remains consistent with the 3–5 W device power budget.

Before turning to test behavior, we note that both pipelines train perfectly on the clean data: optimization is stable, validation accuracy increases smoothly, and no signs of divergence or overfitting are observable in the curves. This apparent success is precisely why we evaluate on augmented inputs; the augmented test set reveals how much of the in-distribution performance carries over under realistic degradations and highlights where the two designs differ once conditions deviate from training. On the augmented test set two effects are clear. First, crops with similar symptoms show lower global accuracy in the per-class table, indicating that the single-head model mixes cross-crop patterns under degradations. Second, the hierarchical pipeline raises accuracy within most crops, consistent with specialization when decisions are confined to a single crop; this appears as higher per-crop values in Table 2. Training–validation curves typically look smoother for the standard learning due to maximal pooling of heterogeneous data, whereas the hierarchical pipeline converges quickly but to crop-dependent plateaus that reflect varying difficulty under augmentation. Notably, these experiments were performed using both FedAvg and FedProx. However, the outputs were almost identical.

When comparing the two pipelines end to end, three observations emerge that align with the anticipated trade-offs. First, the standard learning classifier achieves competitive overall accuracy on the augmented dataset and demonstrates clear advantages in computational efficiency. It requires only a single forward pass per image, consumes less peak memory, and exhibits lower latency and power consumption per image, as shown in the device-level summary at Table 3. Second, the hierarchical approach delivers superior disease-recognition performance for most crops, reflected in consistently higher per-crop accuracy values (Table 2). However, this accuracy gain incurs additional computational cost: each inference invokes both the crop-routing module and a crop-specific disease classifier, resulting in increased latency, memory usage, and energy consumption. Moreover, although the individual crop-specific heads are lightweight, the collective storage footprint across all heads is larger than that of the single-model baseline. Finally, across hardware platforms, the GPU-enabled (Jetson-class) device reduces inference latency for both pipelines but operates at higher power levels, whereas the CPU-only (Raspberry-class) device generally exhibits lower instantaneous power draw and memory usage at the cost of reduced throughput. These differences are exacerbated in the hierarchical pipeline due to its two-stage inference structure.

A crucial consideration is routing. In the experiments, the per-crop disease heads outperform the single global model in almost every crop, and the crop classifier is sufficiently accurate that the end-to-end per-crop pipeline exceeds the global model’s accuracy on nearly all crops, sometimes by a large margin. This confirms the benefit of specialization: when the router is reasonably accurate, constraining decisions within a crop produces higher robustness under the augmented test conditions. The cost remains higher, with two forward passes and more parameters in storage, but when accuracy is the primary constraint in realistic field imagery, the observed gains justify the additional latency, RAM, and energy. Moreover, in deployments with fixed crop identity, bypassing the router and deploying only the relevant head yields the best of both worlds: higher accuracy than the global model and lower runtime cost than the full per-crop pipeline.

Despite these gains, the global model remains useful. Routing introduces an extra point of failure, and its errors compound multiplicatively with the disease head; under harsher degradations than those in our augmented set (e.g., severe motion blur, extreme compression, heavy occlusion), both routing and within-crop classification would be expected to drop, potentially pushing the per-crop pipeline below the global model even if each component remains individually competitive. At the other extreme, when imagery closely matches the clean training distribution, the global model approaches the same “near-perfect” plateaus while retaining clear advantages in simplicity, latency, and energy, which is attractive for single-model maintenance on resource-limited devices. In short, the per-crop pipeline is preferable for typical field conditions where accuracy is at a premium, while the global model is a pragmatic choice for constrained deployments or for scenarios with either very clean or extremely degraded inputs where avoiding multiplicative routing errors, and minimizing resource use can be decisive.

## 7. Discussion and Practical Applicability

In this work, our goal is to isolate and compare the learning behavior of the standard and hierarchical FL pipelines under controlled networking conditions, focusing on model—level performance, communication costs and energy consumption. For this reason, we adopt a fully synchronous FL configuration and ideal environmental and networking conditions to eliminate confounding factors related to client availability. To this end, we allow a fair and reproducible comparison between the two proposed pipelines. This assumption is noted in Section 5.2, where each round requires participating clients to send their updates before aggregation takes place.

To outline the practical limitations of synchronous FL and possible mitigation strategies, we highlight the following:

1. Client dropout and intermittent connectivity are common in open field deployments, due to weather signal degradation, long communication distances and low-power sleep schedules of edge devices.
2. Synchronous FL may stall when waiting for all clients, reducing practicality in scenarios with unstable links.

Potential solutions, which can be incorporated in a future work extension of the present work, may include the following:

- Lightweight error-tolerant protocols for nodes with constrained energy budgets.
- A client selection strategy (or mechanism) that prioritizes nodes with stable connectivity. Additionally, possible rotation selection to improve robustness.
- Semi-asynchronous aggregation policy, which allows the ECN to update global models using available client updates without taking into consideration offline nodes.

One of the key takeaways of our work is the performance gap between the standard learning and the hierarchical learning FL pipelines. In our experiments, the hierarchical learning outperforms the standard learning method by 0.9 on average in terms of average F1-score. But this performance comes with some trade-offs. The first trade-off considers power consumption, which is substantially higher in the hierarchical learning pipeline. The two-model inference procedure consumes 1.6× to 1.8× more power compared to the single-model inference operation, which is conducted by the standard pipeline. The second trade-off is related to the memory (RAM) requirements of the inference, which are essentially doubled for the hierarchical learning pipeline. The third trade-off considers the model management costs, which are substantially more demanding when deploying the hierarchical learning pipeline. This holds true since hierarchical learning requires the maintenance of 1 crop classification model and of *N* disease classification models, where *N* is the total number of plant diseases covered by the system. This increases the system complexity, especially when a large number of IoT nodes are involved. The fourth trade-off is the high model retraining costs of the hierarchical deployment. In real-world applications, the AI models should be retrained to either increase their accuracy, or to account for additional classes. This task is significantly easier when a single model is utilized and more complex when several models are deployed. Evidently, despite the performance advantages of the hierarchical learning, the decision of which pipeline to deploy is a design choice that depends on the requirements of the application under examination.

## 8. Conclusions

This paper presented a distributed deep learning framework based on federated learning (FL) for the diagnosis of plant diseases in IoT-enabled sensor networks. The proposed architecture integrates a two-layer system comprising multiple IoT sensor nodes and an ECN that coordinates model aggregation and synchronization. Each IoT node independently collects plant and environmental data and trains a local DL model, while the ECN aggregates these local models to form an improved global model. This distributed learning approach enables efficient use of computational and power resources, enables large-scale deployment, and maintains consistent model performance across different agricultural environments.

To extend to other agricultural applications, we emphasize that our model architecture is *task-agnostic*. In essence, we state that the system is compatible with any DNN architecture and can accommodate different classification tasks. Moreover, the FL pipelines support multi-class extensions, making them suitable, not only for plant disease classifications, but also for other vision-based agricultural tasks. These tasks may include weed identification, pest detection and crop-yield prediction. Finally, the system architecture already accommodates heterogeneous sensing modalities, enabling future integration of environmental, spectral or multi-sensor inputs.

## Figures and Tables

**Table 1:** The hyperparameters chosen for the FL pipelines.

Parameter	Value
Loss function	CrossEntropyLoss (label_smoothing = 0.1)
Optimizer	Adam
Learning rate	$1\times 10^{-4}$
Weight decay	$1\times 10^{-4}$
Batch size	32
Training epochs	5
Training rounds	10
Clients	10

**Table 2:** Augmented test set results for both pipelines.

Crop	Global Acc	Per-Crop Acc (CropAcc × DiseaseAcc)	Global F1	Per-Crop F1
Apple	0.771	0.807 (0.950 × 0.850)	0.664	0.821
Blueberry	0.890	0.933 (0.933 × 1.000)	0.785	0.966
Cherry	0.709	0.842 (0.916 × 0.920)	0.638	0.867
Corn	0.852	0.723 (0.975 × 0.741)	0.762	0.724
Grape	0.730	0.666 (0.879 × 0.758)	0.791	0.685
Orange	0.897	0.952 (0.952 × 1.000)	0.840	0.976
Peach	0.736	0.866 (0.976 × 0.887)	0.823	0.853
Bell Pepper	0.577	0.659 (0.693 × 0.951)	0.667	0.770
Potato	0.599	0.501 (0.718 × 0.699)	0.726	0.565
Raspberry	0.830	0.920 (0.920 × 1.000)	0.899	0.958
Soybean	0.734	0.892 (0.892 × 1.000)	0.809	0.943
Squash	0.766	0.718 (0.718 × 1.000)	0.792	0.836
Strawberry	0.729	0.814 (0.903 × 0.901)	0.817	0.840
Tomato	0.660	0.642 (0.924 × 0.694)	0.650	0.661

**Table 3:** Latency, power, and memory by device and pipeline per image.

Device	Processing Time (ms)		Average Power per Image (W)		Peak RAM (MB)	
	Standard	Hierarchical	Standard	Hierarchical	Standard	Hierarchical
Raspberry-like	4.267	7.694 (4.24 + 3.454)	0.003556	0.006412 (0.003533 + 0.002878)	894.199	1831.443 (915.721 + 915.722)
Jetson-like	4.379	7.698 (4.238 +3.46)	0.006082	0.010692 (0.005886 + 0.004805)	859.27	1847.715 (923.855 + 923.861)

## Data Availability

The original contributions presented in the study are included in the article; further inquiries can be directed to the corresponding author.
